# Effects of Air Splints on Sensorimotor Disturbances of the Affected Upper Extremity and Trunk Control in Adult Post-Stroke Patients

**DOI:** 10.3390/jcm14155185

**Published:** 2025-07-22

**Authors:** Ana Isabel Useros-Olmo, Roberto Cano-de-la-Cuerda, Jesús Rodríguez-Herranz, Alfonso Gil-Martínez, Alicia Hernando-Rosado

**Affiliations:** 1 Hospital Beata María Ana, Unidad de Daño Cerebral, 28928 Madrid, Spain; anaisabel.useros@fundacionhospitalarias.org (A.I.U.-O.); jrodriguezh.hbma@hospitalarias.es (J.R.-H.); 2 Department of Physiotherapy, Centro Superior de Estudios Universitarios La Salle, Universidad Autónoma de Madrid, 28023 Madrid, Spain; alfonso.gil@lasallecampus.es; 3 Motion in Brains Research Group, Instituto de Neurociencias y Ciencias del Movimiento (INCIMOV), 28023 Madrid, Spain; 4 Department of Physiotherapy, Occupational Therapy, Rehabilitation and Physical Medicine, Facultad de Ciencias de la Salud, Universidad Rey Juan Carlos, 28922 Madrid, Spain; 5 Laboratorio de Análisis del Movimiento, Biomecánica, Ergonomía y Control Motor (LAMBECOM), 28922 Madrid, Spain; 6 Physiotherapy Unit, Hospital Universitario La Paz-Carlos III, IdiPAZ, 28029 Madrid, Spain; 7 Cranio Spain Research Group, Centro Superior de Estudios Universitarios La Salle, Universidad Autónoma de Madrid, 28049 Madrid, Spain; 8 Department of Physiotherapy, Universidad Alfonso X el Sabio (UAX), 28691 Madrid, Spain

**Keywords:** air splints, physical therapy, rehabilitation, sensorimotor impairments, stroke, upper limbs

## Abstract

**Background:** The present study aimed to determine whether the protocolized use of pneumatic splints within neurodevelopmental therapeutic approaches produces a positive effect on sensorimotor impairments of the hemiplegic upper extremity in patients. **Methods:** A randomized clinical single-blind trial was conducted. Stroke patients were recruited and randomized into an experimental group, which completed a treatment protocol of splinting plus physiotherapy for 45 min per session, two sessions per week for four weeks; or a control group, which received the same type of conventional physiotherapy treatment for the same period of time. The patients were evaluated by Fugl-Meyer Assessment of the Upper Extremity (FMA-UE) and the Trunk Control Scale. Secondary variables were Mini-BEStest, the modified Ashworth scale for ankle flexors, and computerized measurements of upper limb functional parameters performed by Armeo Spring^®^ robotic systems and Amadeo^®^. All variables were measured pre- and post-treatment. **Results:** Twenty stroke patients with subacute and chronic stroke completed the protocol. Mann–Whitney U tests showed statistically significant differences between groups for the FM sensation variable (Z = −2.19; *p* = 0.03). The rest of the variables studied in the comparison between the two study groups did not present statistically significant differences (*p* > 0.05). **Conclusions:** The use of air splints in combination with physiotherapy treatment produced improvements in exteroceptive and proprioceptive sensitivity in post-stroke adult patients in the subacute and chronic phases.

## 1. Introduction

One out of two patients experience decreased sensation in the upper extremity (UE) after stroke, which has a direct impact on functional capacity and quality of life [1]. Sensorimotor disorders, which include specific alterations in exteroceptive and proprioceptive sensitivity, hinder motor control because such deficits play a crucial role in motor recruitment for generating force. Proprioception, an essential mediator, is crucial for more precise and efficient motor control [2].

Repeated activation of sensory inputs through passive therapeutic interventions has shown a beneficial effect on cortical motor plasticity and alterations in somatosensory function [3,4]. Among the passive therapeutic interventions used in the field of functional neurophysiotherapy, the use of continuous pressure pneumatic splints (CPPS) or air splints can be implemented as a tool that provides a more enriched therapeutic environment to promote the integration of the affected limb during active movement, thereby limiting the phenomenon of learned non-use [5,6].

The splint material is typically transparent and composed of a double layer of flexible PVC (according to European standards). These splints are designed to be inflated orally with a maximum pressure of 40 mm Hg, which enables the application of deep pressure to stabilize the different articular segments, keeping them in correct alignment during movement [7,8,9,10]. This generated alignment favors not only static postural control but also dynamic control, which could promote improvement in trunk stability, based on the development of activities that emphasize the recruitment of low load within the execution of movement patterns and engrams with repeated use of the affected limbs [11].

Nonetheless, integration of the use of air splints to prevent disuse and disability secondary to stroke, through early activation of the affected limbs, is not systematized in hospitals or the home. Therefore, it is important to evaluate the effects of these devices during the acute phase of the rehabilitation process, integrating the theory of dynamic control systems and motor learning based on the available evidence on the importance of multimodal sensory training of patients [12,13,14].

The aim of this clinical trial was to determine whether the protocolized use of CPPS, applied in combination with problem-solving-based treatment (neurodevelopmental therapy), could have a positive effect on sensorimotor alterations of the affected UE, as well as on motor control of the trunk and balance, in post-stroke patients. Secondarily, we aimed to analyze the effects of CPPS on the functional active movement of the affected UE in the frontal, sagittal and transverse planes, as well as on the strength of the hand musculature and the active range of motion of the fingers.

## 2. Material and Methods

### 2.1. Study Design

A single-blinded randomized clinical trial was conducted (NCT07029061), which included patients who had experienced a stroke at least two months earlier and were receiving treatment at the Brain Injury Unit of the Beata María Ana Hospital (Madrid, Spain). The clinical trial was conducted and reported in strict accordance with the Consolidated Standards of Reporting Trials (CONSORT) guidelines.

### 2.2. Subjects

Participants were randomized into two groups: an experimental group (EG) and a control group (CG). Randomization was performed using the Graphad^®^ program V.8. Two investigators, blinded to the interventions, summoned patients for initial assessment. Thereafter, both groups received treatment for a period of four weeks. Subsequently, the same blinded evaluators performed the final evaluation.

The randomized selection of patients was carried out among individuals in the early and late subacute phases of treatment (from two to six months post-onset), as well as among patients in the chronic phase with stabilized sequelae (from six months post-onset onward) [1,5,6].

The present study received approval from the Clinical Research Ethics Committee of Hospital Universitario La Paz (Approval PI-3867, 20 December 2019). Informed consent was obtained from all patients.

### 2.3. Eligibility Criteria

The inclusion criteria were: (1) individuals who had suffered a stroke at least two months earlier; (2) presenting mild to moderate hypertonicity with a score ≤ 3 according to the modified Ashworth scale in the upper and/or lower extremity (the justification lies in the fact that with spasticity scores above three on the modified Ashworth scale, biomechanical alignment of the upper limb cannot be assumed, making it impossible to properly position pneumatic splints); (3) presence of proprioceptive and exteroceptive alterations that condition motor recruitment in the affected extremities using the Fugl-Meyer Assessment for the upper limb; (4) existence of mild to moderate deficits in trunk stability (trunk control test < 23 and > 8 points).

The exclusion criteria were: patients with (1) traumatic, (2) hypoxic or tumor etiology; (3) patients in a phase of clinical instability with frequent hospital readmissions; (4) wounds or impaired skin integrity of the affected extremities; (5) evidence of structured deformities in the affected extremities; (6) botulinum toxin infiltrations in the last three months prior to the intervention; (7) inability to sit unassisted for at least 30 s; (8) achieving the maximum score on the Trunk Control Test at the time of recruitment (score equal to 23); (9) the presence of language comprehension or cognitive-behavioral impairments as well as perceptual impairments that limit participation; (10) the presence of cerebellar disorders and tremor.

### 2.4. Intervention

#### 2.4.1. Experimental Group

**Figure 1:**
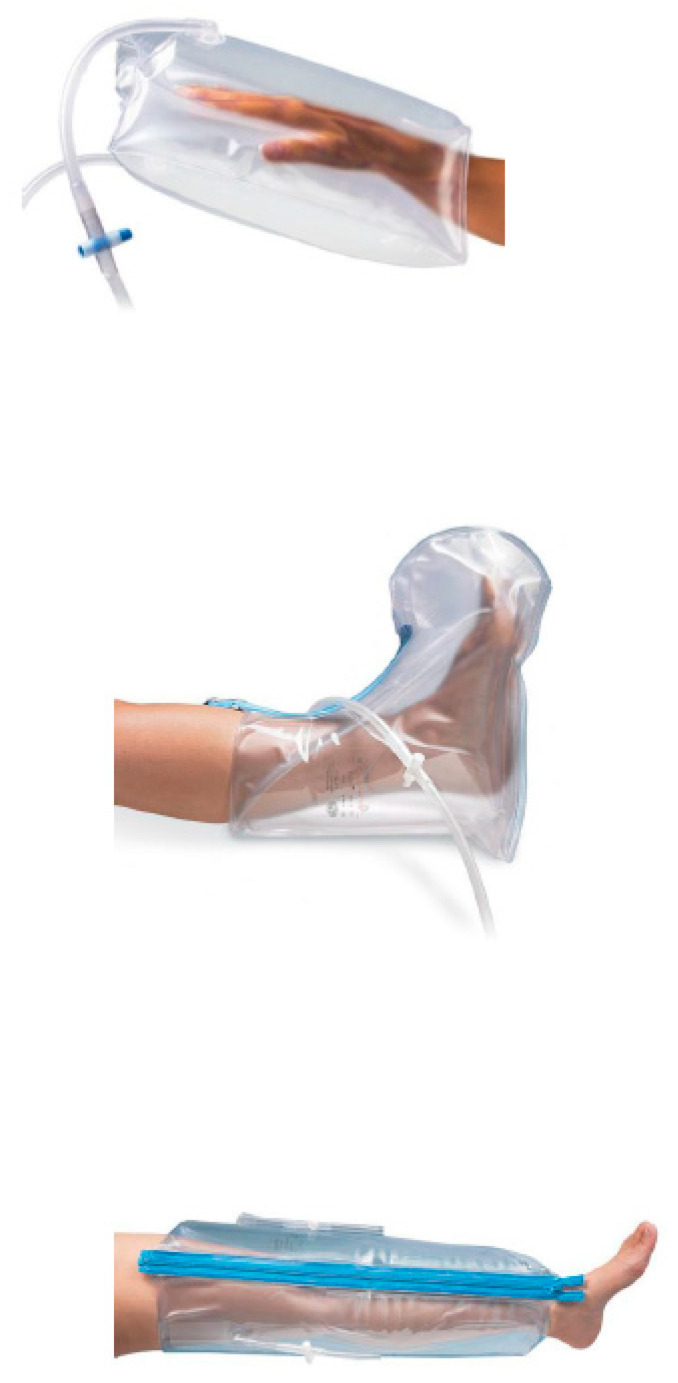
Urias–Johnstone type pneumatic splints.

The EG had CPPS applied within a protocol of activities based on low-load recruitment in closed kinetic chain and correct alignment of the affected limbs, applied together with physiotherapy treatment. Urias–Johnstone air splints (Figure 1) were used. These splints were specifically developed and designed for the treatment of neurological patients with moderate to severe motor control deficits. The choice of splint was established based on the level of motor recovery, the capacity for activation and the specificity of the selected task.

The EG received two 45-min sessions per week. In these sessions, together with physiotherapy treatments, air splints were applied to the affected extremities. The physiotherapy session consisted of the same activities as the CG. Additionally, within the session and for a period of 20 min, the splints were applied to the affected upper extremity, considering the patient’s proprioceptive deficits. In the lower limb, pneumatic splinting of the knee was applied to those patients who presented a knee stabilization deficit which affected the ability to bear weight on the leg, according to the following procedure: selective contraction of the transversus abdominis (TrA) was taught in upright seated position while breathing through the mouth; definition of neutral position in the seated position; low load recruitment in the upright seated position with elevation of the non-affected lower limb and closed chain weight bearing of both hemiparetic limbs, five repetitions involving 10 s of weightbearing; transfer from sitting to standing and from standing to sitting with control of neutral position and eccentric quadriceps work with upper limb support in the intermediate position for 10 repetitions; controlled knee flexion and extension in standing position with elevation of both upper limbs while holding a ball between both hands; concentric and eccentric control of the trunk in standing position with a posterior reference at the level of the pelvis, five repetitions with 10 s of weightbearing; single leg standing support (forward and sidestep) with maintenance of neutral position and selective activation of TrA; exercise using the wall with trunk control and selective activation of TrA, semi-flexion of the knees while the upper extremities work in extension holding a ball for five repetitions.

#### 2.4.2. Control Group

The CG received two weekly 45-min sessions of physiotherapy treatment: trunk alignment training in seated position with midline control for a period of ten minutes; reaching and vertical tracking activities with the affected upper limb for five minutes; activation exercises of the transverse abdominis in an elevated sitting position with anterior trunk displacement along the axial axis for five minutes; maintenance of the elevated sitting position with elevation of the unaffected lower limb for ten minutes; and closed kinetic chain exercises with hand and forearm support for ten minutes. In addition, interventions were carried out seeking functional improvement, repetition of task-oriented activities, movement and control of the pelvis and trunk, motor control activities of both affected limbs, as well as standing activities and gait training.

Both groups received the same total amount of treatment time per session over the four-week period. At home, patients in both the experimental and control groups remained in a standing position for one hour per day, seven days a week, throughout the four-week duration of the study. The experimental group used the splints during standing. The home-based treatment was always supervised by a family member who monitored adherence to the protocol. Adherence was complete in all cases.

A per-protocol analysis was performed rather than an intention-to-treat analysis, as all patients assigned to each group were analyzed, making missing data management unnecessary.

### 2.5. Outcome Measures

Data related to demographic data (age, sex, brain lesion area, stroke laterality) were collected. Also, the following outcome measures were used as primary variables: Fulg–Meyer Assessment of UE (FMA-UE) [15]; Trunk Control Scale [16]. Secondary variables: Mini-BESTest [17]; modified Ashworth scale for ankle flexors [18,19]; computerized measurements of upper limb functional parameters performed by Armeo Spring^®^ (Movart, Volketswil, Switzerland) robotic systems and Amadeo^®^ (Tyromotion, Graz, Austria) [20,21], through functional range of motion (ROM) of the shoulder in the frontal plane of motion measured in meters, functional ROM in the sagittal plane of motion measured in meters, functional ROM in the transverse plane of motion measured in meters, percentage of active finger ROM, active flexion strength of each of the fingers measured in newtons, and active extension strength of each of the fingers measured in newtons.

The FMA-UE [15] is a widely used and highly recommended stroke-specific, performance-based measure of impairment. It is designed to assess reflex activity, movement control and muscle strength in the upper extremity of people with post-stroke hemiplegia. The FMA-UE scale comprises 33 items, each scored on a scale of 0 to 2, where 0 = cannot perform, 1 = performs partially and 2 = performs fully. It is free, requires only household items for testing, and takes up to 30 min to administer. FMA-UE scale scores < 31 correspond with ‘no to poor’ upper extremity capacity, while 32 to 47 represent ‘limited capacity’, 48 to 52 represent ‘notable capacity’ and 53 to 66 represent ‘full’ upper extremity capacity. 

The trunk control test [16] can be used to measure the level of functional motor disability after stroke. The test has prognostic value in relation to the score obtained in the test considering the time of evolution from the date of injury. Likewise, a correlation can be established between trunk control and gait function. The minimum score is zero and the maximum is 100. If the score obtained six weeks after the injury is equal to or higher than 50, it predicts global recovery for gait recovery close to 18 weeks.

The Mini-BESTest [17] is an abbreviated version of the balance test (BESTest), which includes four of the original six sections of the BESTest: anticipatory postural adjustments, reactive postural control, sensory orientation and dynamic gait. The Mini-BESTest contains 14 items in total and the maximum score is 28 points. Each item is scored from 0 to 2 (“0” indicates the lowest level of functionality and “2” is the highest level of functionality).

The modified Ashworth scale [18,19] is a scale that grades tone from 0 (no increase in tone) to 4 (stiff limb in flexion or extension) and has proved to be reliable in adults (in its modified version) for both elbow flexor spasticity and plantar flexor spasticity.

Computerized measurements including ROM and muscle strength, in the affected upper extremity, specifically the shoulder and fingers were measured by Armeo Spring^®^ and Amadeo^®^.

The Armeo Spring^®^ [20,21] device is an exoskeleton used for active treatment of the UE. To use it, active motion is required; it does not provide passive treatment. The Armeo Spring^®^ allows six movements: three shoulder movements (flexion–extension, limited flexion to 90°, abduction–adduction, and external–internal rotation), one elbow movement (flexion–extension), one forearm movement (pronation–supination) and one wrist movement (flexion–extension). In addition, it includes a pressure sensor in the handle, which the user must press during exercises to grasp virtual objects (Kwakkel, Kollen & Krebs, 2008) [21]. The following assessments are available: active ROM for each joint and specific movement (A-ROM), global work area of the MS (A-MOVE), and accuracy + speed of response to a stimulus (A-GOAL).

The Amadeo^®^ device is a robotic system designed for active, passive, and assisted treatment of the hand [22]. Its main purpose is to perform repeated pincer grip and grasping movements. Amadeo^®^ enables the evaluation of the percentage of active range of motion, compared to the passive range of motion of the hand, as well as assessment of the extension and flexion strength of each finger.

### 2.6. Statistical Analysis

SPSS v27 statistical software was used for data analysis. Due to the low sample size and the non-normal distribution of the data for some of the study variables, nonparametric tests were used. Qualitative variables are presented as frequencies and percentages and quantitative variables are shown as medians [interquartile ranges]. The Mann–Whitney U test [23,24,25,26], based on averages and sum of ranks, was used to compare independent groups. The Z statistics represent the effect size of the difference between the study groups. Statistically significant differences were set at *p*-value < 0.05 and the 95% confidence interval was determined.

## 3. Results

### 3.1. Descriptive Analysis

**Figure 2:**
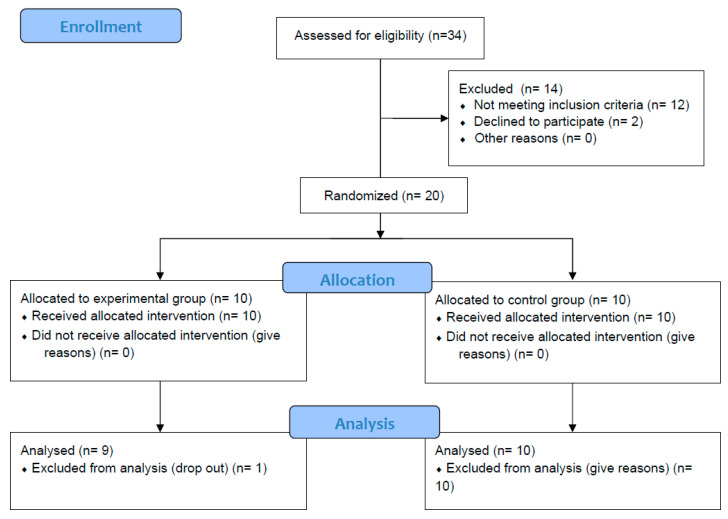
Flowchart.

Twenty adult patients with stroke of vascular etiology in the subacute and chronic phase presenting sensorimotor alterations of the affected upper extremity, as well as low-load motor recruitment deficits at trunk and pelvic stabilizers levels, were recruited (Figure 2). The final sample consisted of 19 participants with a global median age of 63 (range 54–67). Descriptive data of the sample are shown in Table 1. There was only one loss in the study, prior to the process of random assignment to the corresponding group, due to dropout. There was no need to perform intention-to-treat analyses since 100% of the subjects initially assigned to groups were finally analyzed. No adverse events were identified in any of the study groups.

There were no statistically significant differences in the laterality variable in the comparison between groups (Chi^2^ = 0.04; *p* = 0.85).

#### 3.1.1. Comparative Analysis of Independent Measurements

The Mann–Whitney U test showed statistically significant differences between the groups for variable FM sensation (Z = −2.19; *p* = 0.03) (Table 2).

#### 3.1.2. Amadeo^®^

There were no statistically significant differences between CG and GE in hand flexion strength (Z = −0.16; *p* = 0.8), hand extension strength (Z = −0.12; *p* = 0.9), or amplitude (Z = −0.57; *p* = 0.56).

#### 3.1.3. Armeo Spring^®^

Statistical analysis showed no statistically significant differences between CG and GE in frontal (Z = −1.82; *p* = 0.07), sagittal (Z = −1.61; *p* = 0.11) or transversal (Z = −1.6; *p* = 0.11) active motion.

The remaining variables studied in the comparison between the two study groups did not show statistically significant differences (*p* > 0.05). All data of comparisons between the quantitative variables are shown in Table 2.

## 4. Discussion

The results of the present pilot randomized clinical trial show that the use of CPPS, applied for a period of four weeks within a protocol of activities based on low-load stability with integration of the affected limbs in correct alignment in adult patients after stroke, increases both exteroceptive and proprioceptive sensitivity in the upper limb, measured using the FMA-UE scale [15,27,28,29]. This clinical assessment tool has proved to be effective for the evaluation of sensorimotor function, with excellent consistency, responsiveness and good accuracy in the application. All patients belonging to both the test and control groups had dysfunction in the affected upper extremity as measured by the FMA-UE scale. This correlates with the involvement of the middle cerebral artery, which is affected in three-quarters of adult post-stroke patients, resulting in the involvement of the aforementioned limb [30].

The activities structured in the EG intervention protocol, together with the application of the program with pneumatic splinting of the affected limbs for four weeks, emphasized motor and sensory stimulation within the neurophysiological treatment approaches, thus increasing sensorimotor stimulation based on the therapeutic modality introduced by Johnstone [31,32,33,34]. The deep pressure provided by pneumatic splints increases stability in the alignment of the affected upper extremity and inhibits reflex muscle activity, according to studies published by Margaret Johnstone et al. [35]. These authors observed that the application of splints 30 min before exercise showed an inhibition of hypertonicity with increased sensory input due to the activation of Golgi bodies in the tendon organs [36]. The present study attempts to evaluate the effects of pneumatic splints on the affected upper extremity in the short term and once they are no longer used in therapy. The use of the splints enables the performance of selective movements in correct alignment of the affected limbs, which promotes motor control in normalized movement patterns [11,37,38].

Initially, the use of air splints was oriented towards decreasing the excitability of the alpha motor neuron in cases of spasticity, especially in neurological patients with spinal cord injury and cerebral palsy, and their effects were proved while maintaining the use of the air splints [39,40]. Currently, studies by Cambier et al. show that the use of intermittent pneumatic compression clinically improves sensory function, applied for a period of four weeks, using the Nottingham Sensory Rating Scale as a measurement tool. The results of our study, in which patients in the control group were maintained within a functional neurophysiotherapy program based on the neurodevelopmental model (problem-solving), confirm that the inclusion of a continuous pressure of no more than 40 mm Hg in the affected extremities improves both the exteroceptive and proprioceptive sensitivity of the affected upper extremity, with no improvement in active or passive range of motion or pain.

In addition, we wanted to objectively evaluate hand strength using robotic devices (Amadeo^®^) by measuring both flexion and extension strength of the fingers and the percentage of active range of motion of finger opening and closing, in relation to passive mobility. Measurement of the functional active range of motion of the affected UE in the frontal, sagittal and transverse planes was carried out using the Armeo Spring^®^ system. No statistically significant differences were obtained in the measurements performed with both devices. In the case of the active range of motion of the affected upper extremity in the frontal plane evaluated with the Armeo system^®^, the *p*-value was very close to statistical significance. The results regarding finger strength and the percentage of active range of motion of finger opening and closing are comparable with those published by Robson et al. [41] in reference to the overall effects of non-pneumatic splints used in the wrist, where they observed that the use of splints in an increased joint range did not prevent loss of active range of motion.

The results obtained appear to correlate with the conclusions of the meta-analysis performed by Lanning et al. [42], where it was observed that the use of pneumatic, continuous-pressure splints could enhance the effects of task-based training, as previously observed by Serrada and Pollock [43,44].

In relation to the research by Harris et al. [45] concerning the importance of the recovery of motor control of the scapula and the improvement in trunk stabilization, as objectives in the present study, we aimed to evaluate the influence of closed chain work of the affected upper extremity using pneumatic splints on trunk control, measured using the Trunk Control Scale. The results of the present study showed significant differences in the test group compared to the control group, since both groups improved within the treatment framework. In our study, no significant differences were observed between the control and experimental groups in Mini-BESTest scores. This finding is consistent with the results of the Trunk Control Test, which also showed no significant improvements. Given the well-established relationship between trunk control and balance, these outcomes were expected. It is likely that the brief duration of the intervention did not lead to meaningful changes in motor recruitment patterns, which may explain the absence of improvements in postural control and balance performance.

The improvement in proprioceptive sensitivity obtained in the affected UE may correlate with a potential improvement in motor function related to increased recruitment as reported in the meta-analysis by Lin et al. [46], which studied the relationship between proprioceptive impairment and motor deficits after stroke. The main conclusions of this meta-analysis indicated that increased proprioception contributes to increased scores in the domains of movement function, activity development and independence, as measured with the International Classification of Functioning, Disability and Health. The active and passive mechanisms that contribute to increased proprioception during treatment may be useful for the design of activities that enable greater activity and use of the affected upper extremity, preventing non-use behavior from the subacute phase of treatment. It is important to consider, despite our findings, the lack of protocolized and systematic integration of air splints in therapy to promote proprioceptive improvement and the prevention of secondary disability and motor control delay, as supported by the studies of Rabadi et al. [47,48,49,50]. The present study is therefore the first to our knowledge to provide a protocol of activities and home use combined with the use of splints in stroke patients.

Finally, the use of CPPS in the treatment of stroke patients may enable the incorporation of repeated and intensive practice, using external verbal stimulation and feedback during both hands-on and hands-off treatment, and can be used with sensory, motor, cognitive and perceptual alterations of varying degrees. The possibility of using air splinting both during the subacute hospital stay and in the chronic ambulatory phase, as well as at home, would substantially increase the daily training time with the guarantee of adequate alignment and avoiding compensation and learned nonuse [51,52,53].

The strengths of the present pilot study lie in the fact that it is a randomized, evaluator-blinded clinical trial. The evaluations were carried out using validated and highly objective systems, such as the Amadeo^®^ and Armeo Spring^®^ robotic systems.

This research presents several limitations. The main limitations derive from the sample size due to the difficulty of complying with the inclusion criteria, considering the recruitment of patients with vascular etiology. Due to the average duration of the rehabilitation process in neurological patients, it was difficult to increase the sample size or to perform follow-up evaluations after medical discharge. In addition, the patients were at different stages in their rehabilitation process, ranging from subacute to chronic phases, and therefore the results were not limited to a specific phase of the post-stroke process. Additionally, since the patients were not blinded, the placebo effect may have biased the results. Furthermore, the absence of a post-intervention follow-up period, together with the lack of long-term retention data, may have led to the loss of valuable information. Future research is needed to expand the sample size and to use tests that are sensitive to possible specific changes in the improvement of the low-load stability of trunk-pelvic stabilizers or of the motor control and stabilization capacity of the scapula within the closed-chain activities designed within the performance protocol of the test group.

## 5. Conclusions

The use of air splints within an activity protocol that emphasizes alignment control and closed kinetic chain activity of the affected upper extremity improves exteroceptive and proprioceptive sensitivity in adult patients with stroke of vascular etiology. These results could justify the use of CPPS as an effective tool in sensorimotor stimulation from the early stages of treatment. The stabilization functions of the CPPS not only enable static alignment but also more efficient dynamic motor control, which promotes a more proactive approach for the patient, limiting learned non-use and enabling better muscle recruitment via proprioceptive stimulation. These findings should be verified in further research with increased sample size, a follow-up period, and more sensitive measurement tools.

## Figures and Tables

**Table 1:** Descriptive statistics.

	CG (n = 10)	EG (n = 9)	*p*
Age (years)	58 [53.7–63.7]	67 [58.5–79]	0.06
Laterality	L: 6/R: 4	L: 5/R: 4	0.84
Left hemiparesis (frequency)	6	5	
Right hemiparesis (frequency)	4	4	

Age expressed as median and interquartile range using the Mann–Whitney U test. CG: control group; EG: experimental group. L: left; R: right.

**Table 2:** Comparative results of independent measurements.

Variables	CG		EG		*p* Values	Z-Value
	Pre Median [Range]	Post Median [Range]	Pre Median [Range]	Post Median [Range]		
Clinical variables. CG (n = 10); EG (n = 9)						
FM motor	28.5 [8–38.5]	34 [9.5–44]	8 [6.5–44]	16 [8.5–51.5]	0.806	−0.245
FM sensitivity	6 [3.7–10]	8.5 [5.5–10]	10 [3–12]	12 [8.5–12]	0.029 *	−2.185
FM passive	19 [13.5–20]	18.5 [15.7–22.2]	17 [14.5–19.5]	20 [18.5–20.5]	0.651	−0.452
FM pain	24 [21–24]	24 [23–24]	23 [17–24]	24 [21.5–24]	0.728	−0.347
TCT	80.5 [58.7–100]	100 [71–100]	74 [62–100]	100 [81–100]	0.741	−0.330
MBT	9 [4.7–18.5]	13 [6–23.5]	0 [0–10.5]	6 [1.5–14]	0.175	−1.357
Amadeo® GC (n = 10); GE (n = 9)						
Hand flexion strength (kg)	1.58 [0.95–3.71]	2.76 [0.9–4.4]	2.6 [0.7–4.5]	2.8 [1.5–5.3]	0.870	−0.163
Hand extension strength (kg)	0.08 [0.02–0.31]	0.17 [0.03–0.65]	0.01 [0.0–0.6]	0.19 [0.02–1.08]	0.902	−0.123
Amplitude (% of total passive mobility)	7.5 [1.7–20.6]	13 [3–51.5]	2.1 [0.1–75]	10 [0.3–85.5]	0.567	−0.572
Armeo^®^ GC (n = 10); GE (n = 9)						
Active motion (frontal)	27 [17.7–52.7]	45.5 [37–60.5]	52.5 [35–71.5]	75 [73.5–83.5]	0.069	−1.818
Active motion (sagittal)	25.5 [13.7–36]	34 [30.7–41.7]	28.5 [23.2–37.5]	43.5 [36.7–46.5]	0.108	−1.609
Active motion (transversal)	37 [18.7–60.5]	65 [57.5–74]	56.5 [45.5–69]	74 [72.2–78.7]	0.109	−1.604

*p* < 0.05 * using the Mann–Whitney U test. FM (Fugl-Meyer Assessment of Upper Extremity); TCT (Trunk Control Test); MBT (Mini-BESTest); CG: control group; EG: experimental group.

## Data Availability

The original contributions presented in this study are included in the article. Further inquiries can be directed to the corresponding author(s).
